# Endpoints in stem cell trials in ischemic heart failure

**DOI:** 10.1186/s13287-015-0143-9

**Published:** 2015-08-29

**Authors:** Marko Banovic, Zlatibor Loncar, Atta Behfar, Marc Vanderheyden, Branko Beleslin, Andreas Zeiher, Marco Metra, Andre Terzic, Jozef Bartunek

**Affiliations:** Cardiology Department, University Clinical Center of Serbia, Belgrade Medical School, 11000 Belgrade, Serbia; Mayo Clinic, Rochester, MN 55905 USA; Cardiovascular Center, OLV Hospital, 9300 Aalst, Belgium; Cardiology Department, Goethe University of Frankfurt, 60590 Frankfurt, Germany; Cardiology Department, University of Brescia, 25123 Brescia, Italy

## Abstract

Despite multimodal regimens and diverse treatment options alleviating disease symptoms, morbidity and mortality associated with advanced ischemic heart failure remain high. Recently, technological innovation has led to the development of regenerative therapeutic interventions aimed at halting or reversing the vicious cycle of heart failure progression. Driven by the unmet patient need and fueled by encouraging experimental studies, stem cell-based clinical trials have been launched over the past decade. Collectively, these trials have enrolled several thousand patients and demonstrated the clinical feasibility and safety of cell-based interventions. However, the totality of evidence supporting their efficacy in ischemic heart failure remains limited. Experience from the early randomized stem cell clinical trials underscores the key points in trial design ranging from adequate hypothesis formulation to selection of the optimal patient population, cell type and delivery route. Importantly, to translate the unprecedented promise of regenerative biotherapies into clinical benefit, it is crucial to ensure the appropriate choice of endpoints along the regulatory path. Accordingly, we here provide considerations relevant to the choice of endpoints for regenerative clinical trials in the ischemic heart failure setting.

## Introduction

With the implementation of rapid coronary reperfusion as a key interventional strategy, the prognosis of patients with acute presentation of ischemic heart disease has dramatically improved [1]. Yet, this success associated with improved survivorship, compounded by the aging of the population, has contributed to an increased prevalence of chronic ischemic heart failure [2]. Indeed, despite multiple treatment regimens, primarily targeting symptom mitigation, the morbidity and mortality of patients with advanced ischemic heart failure have reached pandemic proportions. In an effort to address the root cause of the problem, curative strategies are increasingly being considered. A case in point is the evolution of regenerative medicine technologies aiming to halt or even reverse progressive organ deterioration in the setting of chronic heart failure.

The prevailing unmet clinical need has provided a major impetus for the development of clinically translatable stem cell-based treatment algorithms, which have shown encouraging results in experimental studies. In turn, this has led to a significant international effort in stem cell-based clinical trials. Most clinical trials have focused on stem cell application in acute/subacute ischemic heart disease, targeting prevention of heart failure induction [3]. Collectively, these trials have demonstrated the clinical safety and feasibility of cell-based interventions. However, experience is much more limited in the setting of chronic, florid heart failure [3–5]. To translate the promise of biotherapies into clinical benefit, it is crucial to ensure the appropriate choice of endpoints along the regulatory path of regenerative interventions. These are guided by recommendations of regulatory bodies, such as the Food and Drug Administration (FDA) in the US or the European Medicines Agency, that delineate generic and pathology-specific requirements as well as rigorous criteria of good clinical practice and clinical research. Here, we summarize the considerations relevant to stages and settings of regenerative clinical trials in chronic heart failure.

## Principles, specifics and recommendations for phase I/II heart failure stem cell trials

### General principles

The regulatory and scientific principles and processes utilized in the clinical development of cell therapy are similar to those in more traditional drug trials. The process for generating evidence on the safety and effectiveness of an intervention begins with phase I/II trials which include a limited number of subjects. Pending the feasibility and safety, initial trials may then progress to confirmatory trials. The execution of phase I/II studies is governed by established standards of clinical trials [6] with appropriate trial structure and logistics. They should be based on prospectively declared, detailed protocols written according to ethical standards. It is recommended [7] that, at this stage, measures of success should not be focused on necessarily reaching statistical significance in explorative efficacy signals. Exceptions are safety readouts. They should be the primary endpoint together with feasibility readouts to inform further steps. Like in drug trials, positive phase II stem cell studies with surrogate endpoints do not lead to approval and adoption. They should produce datasets of foundational benefit and safety, justifying subsequent studies with clinically relevant objectives. This stepwise process should ensure that only the safest products with the strongest signals of efficacy move forward to clinical testing in larger populations within definitive phase III trials.

### Specific considerations for design of regenerative trials

Currently, patient-derived stem cells are the primary source for cardiac regenerative therapy [8–10]. Given the specifics of stem cell-based intervention, phase I trials are not meaningful in healthy volunteers and initial feasibility and safety profiles are obtained in a limited number of patients, comparable with other phase I/II trials. At this stage, robust and independently validated pre-clinical information data sets on safety and efficacy should be available and should include stringent product release criteria. Cell product characteristics, such as stability, potency, purity and mechanisms of cell action, as well as cell line production (efficiency, cost, and compliance) should also have been systematically addressed.

Selected previous trials in patients with ischemic heart failure which have included more than 20 patients are reviewed in Table 1. Early experience with cell-based interventions was initially gathered with skeletal myoblasts. This cell type has been abandoned due to safety concerns related to increased risk of cardiac arrhythmias. Ongoing clinical programs typically utilize adult progenitor cells derived from outside the heart (for example, bone marrow) or from the heart, either autologous or allogeneic, in naïve form or guided for optimized efficacy [11]. Traditionally, left ventricular ejection fraction (LVEF) has been the most common surrogate endpoint in these trials. However, its assessment varied among the trials and relied on multiple methods, including transthoracic echocardiography, left ventricular (LV) angiography and cardiac magnetic resonance imaging (cMRI). It is of note that few studies [12] have reported improvement in clinical surrogate parameters without major changes in cardiac function, emphasizing the need for the comprehensive assessment of efficacy signals where the cumulative evidence rather a single endpoint may drive the design of the ensuing clinical translation. Detailed analysis of the field and future leads for clinical translation are provided elsewhere [11].

**Table 1:** Stem cell clinical trials in patients with chronic heart failure

Trial	N	Cell type	Delivery	Timing post-infarction	Primary endpoint
MAGIC37 [39]	97	SM	Epicardial	>4 weeks	No change LVEF
Dib *et al*. [40]	23	SM	Endocardial	>10 years	Improved LVEF and viability
SEISMIC11 [12]	47	SM	Endocardial	Chronic	No change LVEF, improved 6-min walk distance at 6 months
TOPCARE-CHD [41]	121	BM-derived progenitor cells	Coronary	Chronic	Improved survival and decreased levels of NTproBNP and NTproANP in patients who received cells
C-Cure20 [21]	45	Guided cardiopoietic BM-derived MSC	Endocardial	Ischemic cardiomyopathy	Safety/feasibility, improved LVEF by echo and 6-min walk distance
FOCUS-CCTRN [42]	92	BMMNC	Endocardial	>30 days	No changes in LVESV index and maximal oxygen consumption (VO_2max_) by SPECT
POSEIDON [22]	30	MSC	Endocardial	>60 days	No changes in LVEF by echo; improvement in 6-min walk test, MLHFQ in autologous group
Poglajen *et al*. [43]	33	CD34+	Endocardial	>6 months	Increase in LVEF, 6-min walk distance and a decrease in NTproBNP
SCIPIO [44]	33	c-kit + CSC	Coronary	>4 months after CABG	Increase in MRI LVEF after 4 and 12 months and decrease of infarct size after 4 and 12 months
TAC-HFT23 [24]	65	BMMNC, MSC	Endocardial	Ischemic cardiomyopathy	Improved 6-min walk distance, regional myocardial function and decreased infarct size after 1 year (MRI or CT) in MSC group, but not in BMMNC or placebo group

*BM* bone marrow, *BMMNC* bone marrow mononuclear cell, *CABG* coronary artery by-pass graft surgery, *CSC* cardiac stem cell, *CT* computed tomography, *LVEF* left ventricular ejection fraction, *LVESV* left ventricular end-systolic volume, *MLHFQ* Minnesota Living with Heart Failure Questionnaire, *MRI* magnetic resonance imaging, *MSC* mesenchymal stem cell, *NTproANP* amino-terminal pro-atrial natriuretic peptide, *NTproBNP* N-terminal pro-brain natriuretic peptide, *SM* skeletal myoblasts, *SPECT* single-photon emission computed tomography

Relevant to the design is the choice of the delivery method. Each delivery method has its merits and limitations and should consider the cell type under study [13]. Coronary cell transfer relies on cell adhesion during transmicrovascular migration leading to homogenous myocardial distribution. On the other hand, it is associated with low rates of cell retention and cannot be used with mesenchymal cells or skeletal myoblasts due to risk of microvascular obstruction [14]. Direct myocardial delivery using the epicardial or endomyocardial route offer higher rates of retention compared with the coronary mode of delivery [15]. In the current trials, LV endomyocardial injection is the preferred mode of delivery as it can be performed as a standalone procedure. Moreover, modes of enhanced cell delivery are continuously being redesigned [16].

Myocardial engraftment is dependent on a number of additional factors, including local environment, supply of nutrients, immune rejection or co-transfer of supporting cell types, such as fibroblasts or endothelial cells, hydrogels or matrix components [17, 18]. As the current modes of delivery have so far led to suboptimal retention rates [19] or limited long-term cell survival [20], data on retention using specific cell types and methods of delivery should be obtained in the early stages to determine the pharmacokinetics of the given cell product. This, together with the dose-escalation response, should help to devise the optimal delivery strategy, maximizing the chance for efficacy in ensuing trials while retaining the favorable safety profile. Specific consideration is needed regarding the targeting of injections into specifically designated areas when using endomyocardial delivery. On one hand, electromechanical guidance as facilitated by the NOGA® Cardiac Navigation System (Biologics Delivery Systems Group, Irwindale, CA, USA) allows precise location of the dysfunctional but viable myocardium. On the other hand, pragmatic approaches with echocardiography or fluoroscopic guidance of the endomyocardial delivery have demonstrated encouraging efficacy signals within several programs [21, 22], supporting pre-clinical observation of cell migration after myocardial delivery [23]. Standardization of the delivery techniques may help to compare the efficacies of various cell types as indicated from the recent comparison of endomyocardial delivery of mesenchymal and mononuclear cells [24].

The European Medicines Agency has released a statement [25] covering specific aspects related to stem cell-based products for which marketing authorization is being sought. In first-in-man studies, specific safety endpoints may need to be defined based on theoretical considerations and to detect early any toxicity of the final cell product. In cases where sufficient safety evidence cannot be established in the preclinical studies - for example, due to difficulties in finding an appropriate animal model - the evidence should be generated in clinical trials by including additional endpoints for efficacy and safety. The need for and duration of post-authorization long-term efficacy follow-up should also be identified during the clinical trials, taking into consideration results from nonclinical studies and the intended therapeutic effect. In the US, the FDA has postulated that cells or tissues used for therapeutic purposes are codified under the Good Tissue Practice. Consequently, FDA issued guidance about how the biologic and device regulations apply to cellular and genetic therapies [26]. Accordingly, investigational new drug application necessitates detailed study protocols describing the clinical plan as well as the preparation and testing of the therapeutic cell product.

## Endpoints in ischemic heart failure phase I and II trials

General requirements and safety/efficacy profiles for phase I, II and III trials are summarized in Table 2. The primary objective of all initial studies is to establish the safety profile. The European Society of Cardiology Stem Cell Task Group [27] proposed that initial safety readouts should focus on the risk of tissue injury or abnormal growth and the possibility of arrhythmias, requiring Holter monitoring or interrogation of an implantable cardioverter/defibrillator in heart failure. Initial experience should also focus on gathering information on the key pharmacodynamic and pharmacokinetic features of regenerative therapy, which may include assays addressing the mechanistic action, potency, interaction with disease markers, and dependency on the mode of administration. Cell therapy may be associated with challenges in each of these areas that are distinct from traditional pharmacologic therapies. For example, the component of a cell product which contributes to efficacy and from which a dose–response could be established is often unknown, and potency can be quite variable and difficult to quantify. As alluded to earlier, dose dependency of the safety profile should be addressed in parallel to the explorative analysis of the efficacy signals.

**Table 2:** Requirements and safety/efficacy profile recommendations for phase I, II and III trials

	Preclinical, phase I	Phase II	Phase III
Product-regulatory requirements	Kinetics, biodistribution of the cells. Purity, potency and karyotype stability of particular cells. Ensure traceability	Short-term side effects and risk associated with particular cell-based biologics	Performed after preliminary evidence suggesting effectiveness of particular cells
Objective	Safety	Safety/surrogate endpoints	Safety/therapeutic benefit/improved survival
Patient restriction/criteria	Identify target group (safety analysis)	Identify potential responders and non-responders	Include only responders
Sample size	Usually 20 per cohort	From a few dozen to a few hundred	Several hundred or more
Design	Randomized, open label or placebo	Randomized, placebo-controlled	Randomized, double-blinded, placebo-controlled
End-points (feasibility - product and procedure related)	Procedure safety, biological activity of the cells	Safety/feasibility of the procedure, adequate number of cells/dose response	Long-term, substantial evidence of previously observed feasibility/safety
Safety endpoints	Patient tolerance, abnormal cell growth, mutagenesis, tumorigenicity	Patient tolerance, tissue injury, possibility of arrhythmias	Clinically relevant objective: death, clinical events
Efficacy endpoints	Detect surrogate endpoints sensitive to track the therapeutic benefit	1) Further analysis of previously detected surrogate endpoints 2) Exploratory analysis of clinically relevant endpoints	1) Clinically relevant endpoints. Objective (single or composite): improved survival, reduced clinical events/number of hospitalizations. Subjective: symptom score, health-related quality of life 2) Surrogate efficacy endpoints that meaningfully correlate with clinical endpoints

Consistent with the role of phase I/II studies in the regulatory path, the sample size or power of the trial should not be based on efficacy signals but instead on the cohesive spectrum of projected endpoints that are likely to be improved by the intervention or by addressing components of the pathophysiological cascade of heart failure. It is critical not to overemphasize individual outcomes of surrogate endpoints, but rather to look at the totality of evidence they provide about the potential clinical benefit. Given the limited number of patients and focusing on safety/feasibility readouts, positive phase II studies do not lead to approval and widespread clinical use, but instead produce subsequent studies by providing foundational evidence and generating hypotheses. Surrogate endpoints are generally acceptable to evaluate biologic plausibility and feasibility in these early trials, and to aid in hypothesis development for larger phase III studies. Surrogate endpoint parameters used to assess the effects of stem cell therapy on survival in chronic ischemic heart disease should be able to address the safety of cell-based intervention and closely correlate with survival such that changes should reflect changes in the prognosis, and there should be a pathophysiological basis for both relations.

The latter is particularly important as positive results in surrogate endpoint parameters supported by a known pathophysiological basis can be viewed as a probability signal to achieve the later clinical benefit [28–30] and thus provide scientific/mechanistic support to formulate the hypothesis in the pivotal phase III trial [30]. In other words, surrogate endpoints in phase II trials serve as proof-of-concept and provide preliminary evidence of safety and efficacy.

The surrogate endpoint parameters typically used in stem cell trials in chronic heart failure should include endpoints from various domains reflecting target organ changes within the clinical context: measures of LV function and structural evaluation (that is, ejection fraction, end-systolic and end-diastolic volumes and dimensions, pressure-volume relationships, stroke volumes and indexes, ventricular sphericity, infarct scar, perfusion defect, ischemia); biomarkers reflecting the presence and severity of disease (for example, natriuretic peptides, cardiac enzymes, C-reactive protein, cytokines); functional capacity and symptoms relevant to the clinical setting (for example, 6-minute walking distance, maximal O_2_ consumption, ventilatory efficacy/rate of elimination of carbon dioxide slope, New York Heart Association (NYHA) class, angina score); patient-reported outcome, such as quality of life questionnaires, dyspnea and NYHA class alone or in combination.

In these phase I/II trials all surrogate endpoints should be complemented by observational analysis of clinical outcomes related to the given setting and typically include mortality and cardiovascular morbidity, including myocardial (re)infarction, stroke and unplanned hospitalization due to acute heart failure. As phase I/II cell trials are often unblinded, analysis of all-cause safety endpoints (especially all-cause mortality) may reduce possible bias in those trials [30]. In addition to these clinical safety readouts, local assessment of the cell therapy with regard to cell delivery, such as assessment of myocardial damage and myocardial tissue changes, should be part of the global safety assessment. Taken together, phase II studies should aim to demonstrate concordance in surrogate endpoints from different domains and to set the base for the hypothesis in the phase III trial.

## Clinically relevant changes in surrogate endpoint parameters

In ischemic chronic heart failure, the goal is to track the impact on LV remodeling and function. The assessment of ventricular remodeling (that is, characteristic changes in ventricular volumes and wall thickness and shape, ventricular sphericity, pressure-volume loops) could point to positive effects of stem cells in chronic heart failure. As remodeling may lead to a parallel decline in systolic and diastolic volumes, it is possible that LVEF may show only minor, non-significant changes. Changes in LVEF should be evaluated in parallel with other surrogate endpoints such as exercise tolerance or changes in humoral biomarkers. In this regard, lessons should be taken from previous studies addressing the benefits of positive inotropic drugs showing that improved cardiac function did not translate into clinical benefit. On the other hand, clinical benefit may be detected despite no or minimal change in LV function. Thus, the continuation of the clinical path in the early stages is determined by the synergy between the observed surrogate signals and mechanisms leading to this observed improvement. The clinical safety readouts, including mortality, heart failure admissions or occurrence of life-threatening arrhythmia (that is, ventricular tachycardia/ventricular fibrillation), remain the primary safety endpoint. Recommendations for clinically relevant changes in surrogate endpoints in ischemic heart failure trials are given in Table 3.

**Table 3:** Clinically meaningful response in surrogate endpoints in ischemic heart failure trials

Symptoms	Functional domain	LV function remodeling	Quality of life
A change in NYHA class	a) Increase of 50 m in 6-min walk test b) Increase of 10 % in VO_2max_	a) Increase of 5 % in absolute LVEF or; b) Decrease of 20 mL or 20 % (whichever is greater) in LVESV c) Decrease in NTproBNP by 35 % or 300 pg/ml, whichever is greater	a) Improvement in MLHF-Q ≥10 b) Improvement in KCCQ ≥20

*KCCQ* Kansas City Cardiomyopathy Questionnaire, *LV* left ventricular, *LVEF* left ventricular ejection fraction, *LVESV* left ventricular end-systolic volume, *MLHF* Minnesota Living with Heart Failure Questionnaire, *NTproBNP* N-terminal pro-brain natriuretic peptide, *NYHA* New York Heart Association, *VO*_*2max*_, maximal oxygen consumption

Although the investigators of a phase II study may not be able to predict *a priori* how big the effect will be (for example, a change in LVEF), they certainly should be expected to determine the extent of the effect in the end. In addition, a comparison between observed versus expected changes in different surrogate parameters might be one of the best measures of the success of a phase II trial.

cMRI emerged as the gold standard for evaluation of various surrogate endpoints, including cardiac volumes, perfusion and structure. cMRI with late gadolinium enhancement is a powerful tool to determine the scar burden and viability of the dysfunctional myocardium. It can reliably assess the extent of remodeling and fibrosis, and serve as a suitable tool to monitor myocardial changes after stem cell therapy [31]. Myocardial strain calculated from cMRI tagging is currently regarded as the non-invasive gold standard for assessment of regional function [32]. In addition, cMRI enables analysis of metabolic function, such as detection of high-energy phosphate metabolism and blood-oxygen tension determination using blood oxygen level-dependent MRI [33]. Yet, despite being a key method to track the surrogate endpoints [34], widespread use of MRI may be limited by implanted devices.

## Ischemic heart failure and phase III/IV stem cell trials

Phase III trials are conventionally randomized, placebo-controlled, double-blind and designed to generate definitive conclusions about the clinical merits of the applied method. Pivotal phase III trials are meant to provide crucial facts about whether the intervention meaningfully improved the quality of life and decreased cardiovascular morbidity and mortality in order to support regulatory approval. Phase III trials must be clinically driven, emphasizing hard clinical endpoints starting with all-cause mortality/cause-specific mortality [21]. Then, depending on the clinical setting, endpoints include cardiovascular improvement/deterioration, including re-infarction and need for revascularization, life threatening arrhythmias and worsening heart failure. The choice of either all-cause mortality or cardiovascular mortality is still under debate [35]. While all-cause mortality may appear more relevant from the regulatory perspective, it is associated with random noise, diluting the therapeutic impact in high risk patients such as heart failure patients. In this particular setting, non-cardiovascular mortality is increased due to aging or co-morbidities and cardiovascular mortality may be preferred over the all-cause mortality [35]. Nevertheless, in stem cell clinical trials all-cause mortality should serve as a safety readout and should be directionally concordant or at least neutral with the eventual improvement in cardiovascular mortality. The heart failure population remains the most challenging target for cell therapy also from the perspective of tracking worsening heart failure and hospitalizations [34] due to regional differences in health care policies and local standards in patient management. In addition, not all hospitalizations necessarily indicate worsening heart failure and, at times, can be seen as an opportunity to optimize overall patient management. The Heart Failure Association of the European Society of Cardiology proposed in its consensus to define heart failure hospitalization as at least an overnight stay in hospital caused by substantive worsening of heart failure symptoms and/or signs requiring the augmentation of intravenous heart failure therapy, including inotropes, diuretics or vasodilators, ideally pre-defined in the critical events manual [34]. Similar to endpoints using cardiovascular mortality, tracking non-heart failure hospitalizations is also of interest as their reduction or increase concordant with heart failure admissions provides safety reassurance and strengthens the findings of the cardiac-related endpoints.

To determine the sample size for any pivotal trial of cell therapy requires knowledge of the event rates anticipated in the control group based on the standard of care and an estimate of the reduction likely to be achieved by the intervention based on the surrogate efficacy signals from phase II. For example, in a population with advanced heart failure in which the probability of death or heart failure worsening in the course of a year is 35 %, an intervention anticipated to reduce the event rate by 50 % would require 330 patients for a trial with 90 % power to detect the difference at an alpha of <0.01 %. The number of patients that must be enrolled rises to 1,500 if the intervention is expected to reduce the event rate by only 25 %. In addition, if primary endpoint is all-cause mortality, rather than cardiovascular mortality, the sample size will need to be larger to account for the ‘random noise’ added by other deaths due to associated co-morbidities. Such numbers can be reduced by maneuvers such as defining composite endpoints that include classifying patients into categories of improved, unchanged or worse, or by an adaptive design. Though instrumental in predicting estimated rates of events, the standard of care is a dynamic process and projected event rates based on previous published data may often be different at the time of trial execution. This can hamper the expected enrollment rates or be associated with lower than expected numbers of events, leading potentially to trial termination. So far, these risks are not sufficiently considered in chronic heart failure, but in patients with chronic myocardial ischemia or critical limb ischemia, for example, improvements in the standard of care and technology appear to have reduced the pool of patients previously qualifying for cell therapy interventions.

As in phase II studies, the design and control arm are affected by the clinical setting and delivery technique and earlier findings within specific programs. On the other hand, the quality of blinding at this stage is crucial for the critical objective evaluation of clinical endpoints such as quality of life, as well as endpoints such as re-admissions/re-hospitalizations. The blinding and placebo-controlled design can be ethically challenging in cell trials utilizing direct myocardial delivery. In such cases, sham-controlled procedures coupled with a strict separation of unblinded procedural/operational and blinded clinical teams could be considered as an alternative to the standard double-blinded, placebo-controlled design. Additional challenges for the adjudication of clinical events, such as re-admissions, are differences in local clinical practices and increasing economic pressure causing heterogeneity in the rates of admission for otherwise similar clinical presentation. The timing of obtaining the primary endpoint should also be carefully considered in the context of the target population and expected rates of clinical events. Typically, one-year follow-up is the most meaningful time interval; this timing can be shortened if the patient population is enriched to include patients with advanced disease.

## Composite endpoints in ischemic heart failure phase III stem cell trials

Considering that individual ‘hard’ endpoints often require large trials and lack statistical power when insufficient numbers of patients are included, composite endpoints might provide an acceptable alternative to assess the clinical effect of cell intervention. A composite outcome can prove helpful, enabling early clinical adoption in high risk populations or populations with unmet need, as was demonstrated by the initial cardiac resynchronization therapy regulatory approval. Appropriate *a priori* identification of composite endpoints can increase the statistical precision and efficiency of trials and make them less costly. In addition, a composite outcome may be helpful in situations where it is difficult to decide which outcome to elect as the primary clinical outcome measure by using a combination of various readouts from various domains. However, a caveat should be in place as adding more components should not make interpretation of the results complex. At the same time, essential measures of composite outcome should incorporate mortality, morbidity and patient-reported outcome.

A typical positive example of composite endpoint use in a phase III trial is the CAPRICORN study [36], where an important component of the composite outcome was not substantially modified and the combination of several endpoints proved the superiority of the active treatment. A fundamental condition for composite outcome is that individual outcomes contributing to a composite outcome have to be associated with the primary objective. Likewise, their analysis should be done in the hierarchical order where the most significant clinical outcome cancels out any other outcome that is clinically less significant. Moreover, the limitation in terms of different clinical significance of the observed individual endpoints may be leveled by a pre-defined analytical plan giving varying (hierarchical) weight to the components; for example, cardiovascular death 1, re-infarction 0.5, and target vessel revascularization 0.1.

Though the appropriateness of the assigned arbitrary values to each endpoint could be debated, the methodology of hierarchical composite endpoints can reduce the sample size needed to show efficacy compared with a single clinically related endpoint. The Finkelstein and Schoenfeld analytical plan of hierarchical evaluation may serve as an example [37]. For instance, a trial incorporates various events, such as all-cause death, heart failure events, change in 6-minute walk distance and changes in LVEF or end-systolic volumes. The analysis is based on a hierarchy of endpoints and is constructed by comparing every subject in the active arm with every subject in the comparator arm and assigning +1, 0, −1 scores depending on whether the subject in the treatment arm did better, the same or worse. These scores are assigned at the designated time of the primary readout, for instance, at 52 or 104 weeks. In case of mortality, days alive out of the designated time period or number of heart failure events are counted; using a pre-specified cutoff value, other readouts are categorized as meaningful improvement, deterioration or no meaningful change. Mortality is evaluated first. If both died, the one dying later did better. If only one died, the one surviving did better. If neither died, the next point in the hierarchy is evaluated. The one with less heart failure events did better. If they are similar, lower endpoints in the hierarchy, such as 6-minute walk distance, are evaluated. Subjects tied in all these endpoints will then be evaluated for other endpoints, such as volumes or ejection fraction. In the final step, treatment groups are compared using a test statistic based on the sum of net scores for all subjects.

Hence, the choice of individual endpoints from which to compose the hierarchical analytical tree should be based on several considerations: does the composite endpoint measure the severity of the disease - does it cover its critical components? Can its use solve the medical problem or is it just a statistical convenience? Are the individual parameters valid, biologically plausible and of importance to patients? Are the final results clear and meaningful, do they provide a basis for therapeutic decision, does each endpoint support the overall result and do they indicate/predict improved mortality (based on state-of-the-art knowledge)?

Composite endpoints in heart failure trials with stem cells may include cardiovascular death, re-infarction, worsening of heart failure with repeat hospitalizations or intravenous treatment, target vessel revascularization, stroke, implantable cardioverter device therapy, and heart transplantation. Cardiovascular death includes death resulting from an acute myocardial infarction, sudden cardiac death, death due to heart failure, death due to stroke, and death due to other cardiovascular causes, including unwitnessed death without other cause of death. It is also important to emphasize that a complex combination of objective measures of mortality/morbidity with subjective measures (NYHA class) or mechanistic endpoints (that is, brain natriuretic peptide) are difficult to interpret and thus are generally discouraged.

## Future trends

As our knowledge about cardiac stem cell therapies improves, insight into the significance of patient-specific disease management grows. In many cases it will be necessary to tailor the endpoints to meet the needs of the studied population. In this regard, systematic stratification of patients to adequately match clinical trials will be of paramount importance. Therefore, emphasis should be on delineating acute versus chronic disease and allogeneic versus autologous transplantation, identifying responders and non-responders as well as pre-treatment of co-morbidities to limit modifiable confounding factors; this is especially important for stem cell treatment. In every case, hard clinical endpoints stay the necessity for future clinical cell trials, but standardization of outcome measures, choice of cells and cell manufacturing remain an imposed standard in the trial design. Standards of efficacy need to be established as continuous improvements in standard of care lead to better quality of life and survival; likewise, regional differences in health care policies need to be considered as they may impact the assessment of readouts such as readmissions [25].

Another aspect to consider in the design of trials is the increasing financial cost needed for funding large clinical trials required to demonstrate the added value of newly tested treatments. At the same time, increased socio-economic pressure forces physicians and healthcare providers to shift care towards outpatient care or care in the patient’s home and new endpoints reflecting this change in socio-economic and healthcare policies should be considered. In this regard, endpoints based on socio-economic evaluations using parameters such as benefit expressed in terms of quality-adjusted life years might be of help [38]. In this regard, a Markov decision analysis model to calculate the incremental cost-effectiveness ratio in cardiac stem cell trials might be helpful. Positive results in cost per life-year calculations gained with stem cell treatment compared with no treatment might persuade governments and encourage biotechnology companies to further invest in stem cell trials.

## Conclusion

As initial autologous stem cell-based cardiac regeneration therapy led to a rather rapid translation into early-phase clinical trials, the research has now reached the point where conclusive answers and thoughtful fine-tuning are needed. Phase III, large-scale, multicenter, double-blind (sham- controlled), randomized clinical trials performed under rigorous safety standards are necessary to confirm clinical benefits. In this regard, consensus on clinical endpoints and methodologies used to assess those endpoints are necessary to move forward and critically examine the quality of data gathered in future clinical investigations of cardiac stem cell therapy.

## Abbreviations

cMRI: Cardiac magnetic resonance imaging
FDA: Food and Drug Administration
LV: Left ventricular
LVEF: Left ventricular ejection fraction
MRI: Magnetic resonance imaging
NYHA: New York Heart Association
